# Occupational Dermatitis in a Paint Mixer: Impact of Symptom Neglect and Strategies for Early Intervention and Prevention

**DOI:** 10.7759/cureus.76050

**Published:** 2024-12-19

**Authors:** Joshua A Jogie

**Affiliations:** 1 Faculty of Medical Sciences, The University of the West Indies, Saint Augustine, TTO

**Keywords:** early intervention, irritant contact dermatitis, occupational dermatitis, paint additives, personal protective equipment

## Abstract

A 34-year-old paint mixer presented with mild, localized dermatitis on the forearms, traced to low-level exposure to a paint additive. Immediate interventions, including improved personal protective equipment (PPE) and modified job tasks, led to rapid symptom resolution. This report highlights early identification, appropriate workplace controls, and follow-up measures that prevent long-term complications. The case underscores the importance of prompt, targeted interventions and an organizational commitment to worker health.

## Introduction

Dermatitis is a broad inflammatory skin disease marked by redness, swelling, and discomfort [1]. Allergic dermatitis involves early acute phases where the skin reddens and itches, subacute phases with scaling and crusting, and chronic phases featuring thickened, lichenified skin [2]. Unlike irritant dermatitis, which results from direct chemical damage to the skin’s barrier, allergic dermatitis stems from immune-mediated reactions to allergens [3]. If left untreated, dermatitis can spread, cause persistent itching and pain, and lead to secondary infections and scarring [3]. Addressing risk factors and reducing allergen or irritant exposure early can stop mild cases from progressing into severe forms that interfere with daily activities [3].

Occupational dermatitis is a common inflammatory skin condition caused by exposure to substances in the work environment [1]. It affects workers in many industries, especially those handling chemicals, allergens, or irritants [2]. Early detection and intervention can prevent progression to chronic dermatitis and avoid lost work time [2]. Occupational contact dermatitis arises when irritants or allergens compromise the skin barrier or trigger immune responses [3]. Paint mixers face particular risks due to frequent handling of solvents, resins, and additives [4,5]. Even mild dermatitis can decrease comfort, productivity, and overall quality of life [6].

Despite occupational guidelines and safety training, workers sometimes ignore early mild symptoms, risking chronic complications [7]. Without early management, these lesions can worsen, requiring longer treatment and job changes [8]. Effective prevention depends on proper risk assessment, prompt exposure reduction, and implementation of controls such as improved personal protective equipment (PPE), job rotations, and regular health monitoring [9].

This case involves a 34-year-old paint mixer with mild, localized dermatitis on his forearms traced to a paint additive. Prompt intervention led to resolution within days. The employer’s swift action highlights an approach that prevents a mild condition from escalating and demonstrates how early management, supported by the workplace, can maintain employee health and productivity. This supports current literature showing that early, workplace-based interventions can prevent mild occupational dermatitis from becoming severe.

## Case presentation

A 34-year-old male paint mixer presented with a one-week history of mild redness and itching on his forearms. The timing coincided with starting to work with a new industrial paint containing a specific additive. He had worked in this role for three years without significant skin issues. He noted mild dryness during the last three months but never required medical care.

On examination, well-demarcated erythema and mild scaling were visible on the dorsal forearms. No vesicles, weeping, or severe edema were noted. He reported mild itching but no burning or pain. He denied systemic symptoms such as fever. There were no lesions elsewhere on his body.

A detailed occupational history revealed the recent handling of a new paint formulation. Although he wore cotton overalls and nitrile gloves, parts of his forearms remained vulnerable to splashes or contact while mixing. The forearms were a direct site of exposure.

Irritant contact dermatitis seemed likely given the recent introduction of the new additive and the localized nature of the lesions. Allergic contact dermatitis or frictional causes were considered, but the clear temporal link suggested an irritant process. Because the symptoms were mild and consistent with low-level irritant exposure, patch testing was not performed at this stage. The clinical impression favored mild occupational irritant contact dermatitis.

The workplace responded promptly. The company issued longer nitrile gloves to protect the forearms and provided protective sleeves. They altered the patient’s schedule, rotating him out of the mixing role to limit exposure. They also reviewed mixing procedures to reduce skin contact opportunities.

No systemic medication was needed. The patient applied a fragrance-free moisturizer and a mild topical steroid (hydrocortisone 1%) once daily for three days. Within five days, the redness and itching improved substantially. By one week, his forearms appeared normal.

A two-week follow-up visit showed no recurrence. The patient reported no further itching and appreciated the quick intervention. He continued to wear enhanced PPE and followed improved safety protocols. Over three months of observation, no new lesions developed.

## Discussion

This case highlights the significance of early recognition and immediate intervention in occupational dermatitis. Early-stage irritant dermatitis, if neglected, can progress into chronic and more severe forms that require more intensive treatment and possibly result in lost work time [1,3]. The patient’s mild dermatitis resolved rapidly once exposure was minimized and simple protective steps were taken. Symptoms were tracked using a simple visual scale and patient feedback.

Occupational contact dermatitis involves irritants or allergens that damage the skin barrier [3]. Occupational contact dermatitis makes up around 30% of reported occupational diseases. On average, there are about 0.5-1.9 cases per 1,000 full-time workers each year [10]. In this scenario, the additive likely acted as a mild irritant. Frequent low-level exposures compromise the stratum corneum, facilitating inflammation [3]. Even minimal contact over time can cause subtle skin damage that escalates if not addressed. Early intervention-removing or reducing exposure-allows the skin barrier to recover and prevents chronic inflammation.

Allergic contact dermatitis, which involves a delayed-type hypersensitivity reaction, remains another important consideration. Although not proven in this case, allergic reactions can require more intense allergen avoidance measures [11]. If symptoms had persisted or recurred after interventions, patch testing would have been considered.

Patch testing is typically recommended once initial measures fail and suspicion of allergic contact dermatitis remains. It should not be avoided when persistent or unexplained skin lesions suggest an allergen may be involved. Identifying the allergen can help guide targeted avoidance and improve outcomes [12].

Enhancing PPE was a key element in this successful resolution. Extended gloves and forearm sleeves reduced direct skin contact. Job rotation lowered cumulative exposure. These measures align with recommended occupational control hierarchies that favor engineering and administrative adjustments before relying solely on PPE [13]. A supportive work environment that addresses hazards early can reduce the incidence and severity of occupational skin diseases [14].

Occupational dermatitis can have lasting economic and social consequences if allowed to progress [15]. Chronic lesions may need prolonged treatment and impose psychological stress. Early intervention prevents these outcomes, improving employee well-being and reducing costs associated with absenteeism or compensation claims [16]. Training employees to report symptoms promptly and ensuring a rapid organizational response are central to preventing long-term complications [17].

Follow-up is crucial to confirm that interventions remain effective. Reevaluations ensure no recurrent problems and confirm that workplace measures sustain their protective effect [18]. Ongoing communication between occupational health professionals, workers, and management helps maintain high standards of skin safety and keeps minor issues from becoming major problems [19].

Targeted research can refine risk assessment for specific additives used in paints. Identifying irritant thresholds, standard testing methods, and improved PPE designs will further support prevention efforts [10,20]. Current evidence-based guidelines and best practices for diagnosing and managing occupational contact dermatitis encourage early action and close collaboration between employees and employers [6,9,20]. Stricter chemical regulations in paint manufacturing industries can reduce workplace exposures and improve public health [18].

## Conclusions

This case demonstrates that prompt recognition and immediate workplace modifications can resolve mild occupational dermatitis. A 34-year-old paint mixer’s forearm dermatitis, associated with a paint additive, improved rapidly after enhanced PPE and schedule adjustments reduced exposure. Early medical intervention prevented progression and maintained the worker’s comfort and productivity. This example reinforces the value of proactive prevention, ongoing monitoring, and organizational commitment to employee health.
